# Traumatic Tetanus1Paper read at the January meeting of the Roswell Park Medical Club.

**Published:** 1905-05

**Authors:** Charles Bentz

**Affiliations:** Buffalo, N. Y., Lecturer in Bacteriology, University of Buffalo


BUFFALO MEDICAL JOURNAL.

VOL. XLIV.—LX. MAY, 1905. NO. 10

### ORIGINAL COMMUNICATIONS.

### Traumatic Tetanus.[1]

BY CHARLES BENTZ, M. D., Buffalo, N. Y.,
Lecturer in Bacteriology, University of Buffalo.

TETANUS was recognised and described before the Christian era. In 1884, Nicolaier discovered the bacillus tetani. In 1889, Kitasato obtained it in pure culture. It is now recognised as the cause of tetanus. It is a common saphrophytic organism found in garden earth, street sweepings, putrefying organic material and in the intestinal discharge of herbivorous animals. According to Woodhead, certain savage tribes of Africa and the East Indies use soil that is capable of producing tetanus for an arrow poison.

Tetanus may be defined as "an infectious disease caused by a specific organism, which is localised at the point of inoculation. It is characterised by painful spasmodic contraction of the muscles, referable to the nervous system and with the absence of obvious tissue changes." It is the result of a specific wound infection. All mammalia, including man, are susceptible. The human species is especially so.

MORPHOLOGY OF THE TETANUS BACILLUS.—In its vegetative form it is a slim, straight bacillus, with rounded ends, which may form threads. It is slightly motile. In the spore-bearing form, the spores are located at one end which is swollen, giving the bacillus the characteristic drumstick or pin shape. The spores are very resistant. It is stated that they are killed by moist heat at 100° C. in 5 minutes; by bichloride of mercury 1-1000, in 3 hours; by 5 per cent. carbolic acid in 15 hours. The tetanus bacillus stains readily with solutions of the ordinary anilin dyes, also by Gram's method. It is a strict anäerobe. It may be cultivated

1. Paper read at the January meeting of the Roswell Park Medical Club.

at the room temperature, but better in the incubator. It grows upon ordinary culture media, preferably those containing dextrose, and gives off a characteristic odor. Upon gelatin a colony presents characteristic radiating filaments and looks like a thistle.

Mixed infections are favorable to the development of tetanus. Other organisms require oxygen for their growth; hence, by using up the oxygen renders conditions suitable for the development of this germ. The organisms multiply locally at the seat of inoculation and never enter the blood or deeper tissues. The disease is always contracted through wounds which may be of a trifling character. Clinically, persons having the disease suffer from spasms which start from the point of inoculation and finally become general. The period of incubation varies from one day to several weeks. The shorter this period of incubation in the human subject, the more serious is the disease.

Tetanus belongs to that class of diseases which exhibits the element of poisoning by bacterial products in an extremely marked manner. The symptoms are produced by the poison elaborated, which is probably small in amount, but extremely powerful and rapidly produced. Kitasato found that excision and cauterisation of the point of inoculation in mice failed to save the animal unless practised within an hour after inoculation.

It has been estimated that 1-280 of a grain of pure tetanus toxin would be the fatal dose for a man weighing 155 pounds. This poison has a specific affinity for the ganglion cells of the anterior horns of the spinal cord.

Meyer and Ransom explain the delayed effect of the poison thus: the toxin does not reach the spinal cord through the blood-stream, but travels up the axis-cylinders of the motor cells, from their terminations. The myelin sheath acts as an impervious membrane, and the toxin enters at the end of the neuron, where it is not provided with the sheath. Sensory nerves do not transport toxin. Part of the toxin enters the nerves in the region of the wound, the rest is carried by the blood and lymph to be later taken up by the motor nerves in other parts of the body. Experimentally, if tetanus toxin is injected into the spinal canal, the symptoms appear at once. The toxin produces degenerative changes of the Nissel granules and protoplasm of the motor cells.

CASE I.—J. S., male; age, 38; previous history, good. In June, 1902, sustained a compound comminuted fracture of the right leg. A considerable quantity of dirt was found in the wounds. The patient was advised to have his leg amputated. Operation was declined. Microscopic examination was made of the pus from the wounds. A few short bacilli and cocci were found. About the eleventh day after the injury the patient complained of severe headache and stiffness in the muscle of mastication. Another examination of the pus from the wounds was made, a few drumstick bacilli and many cocci were found. Tetanus antitoxin was injected in 20 c.c. amount in the groin every two hours. The other symptoms developed rapidly, trismus became pronounced. The physiognomy of the patient was distinctive. It was immobile, features were fixed, the forehead wrinkled and the corners of the mouth retracted producing the sardonic grin. There was an increased flow of saliva, which escaped from the corners of the mouth. Next there was rigidity of the muscles of the body and then the spine was bowed anteriorly (opisthotonos). The position of the body was constantly rigid. Convulsive seizures of variable duration occurred, causing patient to groan frightfully. The skin was covered with profuse perspiration. The intellect remained clear until death, which occurred two days after the appearance of the first symptoms. Autopsy was not permitted.

Case II.—P. M., male; age, 42. In October, 1903, sustained a crushing injury of the right foot necessitating amputation of four toes. Street dirt was ground into the wound. About eleven days after injury the patient complained of stiffness of the muscles of mastication. A microscopic examination of pus from the wound was made, a few pin shaped bacilli and cocci were found. The patient suddenly died in convulsions. From these findings and the few symptoms present tetanus was suspected.

Autopsy was permitted. The findings were as follows: body was that of a middle aged man, not remarkable in external appearance, except that rigor mortis was marked and there were bandages on the right foot and around the neck, the latter bloody. On removing the bandage from the foot, the first four toes were found amputated at various joints, covered with dressing, the stump of the second toe black, and some purulent discharge present. Smears and cultures were made from the discharge from the toes and the deeper parts of the wounds. On removing the bandage from the neck a sinus was found on the right side, evidently of long standing, a little below the mastoid process. Cultures and smears were made from this sinus.

The brain and upper part of the spinal cord were removed and examined in all parts, but presented no remarkable features. There were no changes in the cranium. There was a roughening of the upper bones of the spine, of an extent which was impossible to determine. It was most marked in the odontoid process of the axis. There was softening and loosening of the ligaments in the vicinity of the atlas and axis. The odontoid process projected abnormally behind and must have compressed the medulla. This was decided to have been the immediate cause of death. A probe inserted into the sinus in the neck went in the direction of the diseased area, about four inches. The sinus evidently led to this point. Cultures and smears were made from the region. Examination of the internal organs were negative, except an area of tuberculosis of the apex of the right lung.

Subsequent study of the smears and cultures was made. Those from the sinus in the neck and from the diseased vertebræ showed ordinary pus cocci. The process was, however, probably tuberculous in the beginning and was very likely connected with the tuberculous area in the lung. The smears made from the foot showed numerous pus cocci, and bacilli having the characteristic form of tetanus bacilli, with a few bacilli of other forms. Subsequent study of the suspicious organisms in culture showed that they presented the characteristics of tetanus bacilli. Among other points their spores still grew well after being at a temperature of 80° c. for 45 minutes. Minute portions of the cultures killed each of three guinea pigs in about 48 hours, producing muscular spasms. The bacilli grew in the absence of oxygen.

It is my opinion that the man suffered from tetanus; that he had a weakening of the ligaments about the upper part of the spine and the lower part of the skull owing to previous tuberculous disease, and that tetanic convulsions led to a partial dislocation of the axis; the odontoid process compressed the medulla and produced death, probably very quickly.

Case III.—Name, F. D.; year, October 31, 1904. Diagnosis, traumatic tetanus. Nature of injury, stepped on rusty nail which penetrated the foot between fourth and fifth metatarsal bones. Period of incubation, 6 days. Method of administration of antitoxin, subcutaneous and into nerve trunk of affected side. Amount, 100 c.c. Other treatment, bromides, chloral, morphine, and chloroform. Result, death.

Case IV.—Name, L. B.; year, August 17, 1904. Diagnosis, traumatic tetanus. Nature of injury, compound fracture of left wrist. Period of incubation, 6 days. Method of administration of antitoxin, subcutaneous. Amount, 200 c.c. Result, death.

Case V.—Name, A. L.; year, July 21, 1904. Diagnosis, traumatic tetanus. Nature of injury, stepped on rusty nail, which penetrated the foot between second and third metatarsal bones. Period of incubation, 5 days. Method of administration of antitoxin, subcutaneous. Amount, 200 c.c. Other treatment, bromides, chloral, morphine, chloroform. Result, death.

Case VI.—Name, E. S.; year, 1904. Diagnosis, traumatic tetanus. Nature of injury, laceration of foot. Period of incubation, 12 days. Method of administration of antitoxin, subcutaneous. Amount, 330 c.c. Result, death.

The fatality of traumatic tetanus quoted in textbooks gives 70 to 80 per cent. of deaths. About 10 to 15 per cent. recover without the use of the antitoxin. The disease is more prevalent in the hot climates than in the temperate. Wells has found that the curve of deaths takes its upward start in May, reaching its maximum in July, and thence declines towards October. This is accounted for by Fourth of July accidents.

Richter in a collection of 717 cases of tetanus caused by various injuries of war surgery, records 631 deaths or a mortality of 88 per cent., with 40 recoveries, equaling 12 per cent.; of these 40, 13 were of milder variety. Behring gives for tetanus a mortality of 80-90 per cent. The records of the Bürger Hospital at Cologne give for tetanus a mortality of 62.5 per cent. Garrigues gives for 57 cases of puerperal tetanus a mortality of 84-92 per cent. Gowers gives for traumatic tetanus a mortality of 90 per cent. Dean gives for all cases in various London hospitals for a period of 16 years a mortality of 80 per cent.

In 1894 a special committee, appointed by the *British Medical Journal,* came to the conclusion that acute tetanus is incurable and that, although anodynes and hypnotics sometimes afford alleviation, there is known no remedy for it. They also found that, between 1875 and 1892, there occurred in England and Wales, 2,969 deaths from traumatic tetanus and between 1881-1892, 568 deaths from so-called idiopathic tetanus.

Stanton reported that as a result of Fourth of July accidents, 422 cases of tetanus developed and of these only seven recovered, a mortality of 98 per cent. Moschcowitz has collected since the introduction of antitoxin in 338 cases, mortality 43.2 per cent.

It should be noticed that the diagnosis in some of these cases reported is questionable; also, that a much larger proportion of the recoveries from tetanus is published than of the fatalities, so that the mortality percentage should be taken with much allowance. Statistics are as yet too meager to justify a final opinion as to the practical value of serum therapy in tetanus, but it appears to be somewhat useful.

Several diseases can be diagnosticated by making a bacteriological examination of the pus from abscesses. In others, it may reveal the cause and character of the pathological process. There are a few diseases the specific organism of which can be diagnosticated with great probability by the exclusive use of the microscope. They include the ray-fungus of actinomycosis, the bacillus tuberculosis, the gonococcus and the bacillus of tetanus.

PROPHYLAXIS AND TREATMENT OF TRAUMATIC TETANUS.—(1) Thorough cleansing of any wounds, received in barnyards, gardens or other places where the bacilli are likely to be present, also Fourth of July wounds, by vigorous operative measures and the removal of any foreign matter. Free drainage of the wounds and immediate injection of 10 to 20 c.c. tetanus antitoxin into the nerve trunk coming from that part is advised; (2) as a prophylactic measure in Fourth of July cases, the enactment of laws by congress, state legislature and municipalities, prohibiting the sale and manufacture of dangerous toys. Recently, Dolley has succeeded in producing the characteristic symptoms of tetanus (convulsions) by inoculating wads from blank cartridge into rats, guinea pigs and rabbits. Several years ago, my colleague, Dr. Frederick C. Busch, succeeded in cultivating the tetanus bacillus from the wad of a blank cartridge which had produced the wound in a fatal case of tetanus. Inoculation of this culture into rabbits produced tetanus. This, of course, does not prove that the bacilli were originally in the wad; (3) the injection of tetanus antitoxin as soon as tetanic symptoms become manifest. "In the human being the disease has made such headway when discovered and so much poison has already been elaborated that therapeutic success after injection of antitoxin is uncertain." Wells says, "a patient who has just developed symptoms of tetanus is not just developing the disease, but is dying of it;" (4) symptomatic. The use of bromides and other hypnotics. All means to lessen spasms, as plugging ears of patient, darkening the room, and the like. The nutrition of the patient should be kept up.

The morphine-eucain treatment, recommended by Murphy, consists in the aspiration of few cubic centimeters of cerebrospinal fluid by lumbar punctures, followed by injection of 3 to 4 c.c. of a solution containing B-eucain, gr. 1½, morphine sulphatis, gr. ⅓, sodium chloride, grs. 3, aqua distillata, ozs. 3½, into the spinal canal. Injections should be repeated until the desired effect is produced.

The Russians inject strained sterile, freshly prepared emulsions of brain tissue (following the principle of Wasserman). Brain tissue has a specific affinity for this toxin. About 30 c.c. of the emulsion in salt solution is injected into the tissues in the region of the wound.

The Italians use Baccelli's carbolic acid treatment which consists of injecting subcutaneously 1 per cent. carbolic acid solution in sufficient quantities to make 5 grains in 24 hours.

The Matthews or cell catharsis (diuresis) treatment: large amounts of a solution containing sodii chloridi, gr. 55.5, sodii sulphatis, gr. 155, sodii citratis, grs. 51, calcii chloridi, grs. 2, aquae, pints 2, are injected. This solution is injected intravenously, in quantities up to 500 c.c. at each infusion, which should be repeated twice in 24 hours. This method is effective in experimental tetanus.

The best results in the use of antitoxin have been attained by those who injected it into the spinal canal. According to Rogers it is necessary to scratch the cord or the nerve filaments with the point of the needle so that the antitoxin reaches the cord. In any case, it is not a question of the amount of antitoxin used, but the effect.

If a prophylactic injection of antitoxin could be administered in every case in which the occurrence of tetanus seemed at all likely, the disease would probably seldom develop.

I am indebted to Dr. Julius Ullman for the histories of some of the cases given.

### REFERENCES.

Moschcowitz. Studies from the Department of Pathology of the College of Physicians and Surgeons, Columbia University. Vol. VII. 1899-1900.

Moore, V. A. The Pathology of Infectious Diseases of Animals. 1902. P. 185.

Stanton. Journal of American Medical Association. August 29, 1903.

Prophylaxis and Treatment of Fourth of July Tetanus. Journal of American Medical Association. June 18, 1904. No. 1621.

Murphy. Journal of American Medical Association. August 13, 1904. P. 461.

Rogers. Annals of Surgery. September, 1904. Vol. XL. No. 3. Pp. 418-419.

Dolley. Journal of the American Medical Association. February 11, 1905.

### DISCUSSION.

Dr. C. M. Brown: It appears to me when we get a suspicious wound, the easiest way to treat it is to chloroform the patient, thoroughly cleanse and cauterise the wound with carbolic acid, and lightly pack the same with iodoform gauze. At this time, and while the patient is under the anesthetic, the antitetanic serum should be injected. Merck's dry serum dissolved in distilled water is the most convenient form to use and is quite harmless.

I have been very much interested in the Matthews treatment. Prof. Loeb experimented with it on animals under control. He injected three times the toxic dose of tetanus toxin, followed by saline solution, which produced free cell catharsis and diuresis, washing out the toxin from the cells. Results were good, but I believe the method has not, as yet, been tried upon man. I believe it is the consensus of opinion of the best authorities, that the antitoxin, to be of service late in the disease or after the symptoms begin to develop, must be injected directly in the subdural or subarachnoid spaces.

Dr. N. G. Russell: A review of statistics shows that there was a considerable advance made in the care and prophylaxis of this disease before the advent of the antitoxin treatment. Prior to 1782, in one Dublin hospital there were about 2,900 deaths from tetanus to 17,000 births; while in Berlin, in 1870, the death rate from this disease in obstetric practice was only about 12 per 1,000. Since the use of antiseptics this has been reduced so that in 1900 there were only 2 per 1,000.

War statistics show a varying frequency but a steady lowering of the death rate, even before the general use of antiseptics. The disease is still too prevalent, as shown by the number of cases reported in New York city alone. In 1899, I think there were 73 deaths reported.

The mortality has been much lowered since the beginning of the use of antitoxin. Reports show a loss of only 30 or 40 per cent. of cases under antitoxin treatment, while the general average under other treatment is about 60 to 80 per cent.

The best results seem to come from large doses of antitoxin given early, before the beginning of symptoms. No other remedy seems to be of much use.

Could not a diagnosis be made early in a suspected case by making a culture from the wound, heating it to 80° C., and thus isolating the organism? Would the application of ice to the wound inhibit growth? The disease will not develop in the frog unless the animal be kept at a temperature of 37° C.

Dr. F. W. Barrows: In my reading I could find nothing to add to this discussion. I remember watching with anxiety two cases; neither developed tetanus however. One case was a penetrating gunshot wound with fracture of two metacarpal bones, considerable laceration, and extremely dirty. This was a boy 12 years old. Treatment consisted of irrigation with 6 to 8 quarts of hot antiseptic solution, 1 to 6 or 8,000 sublimate, and then lysol. At close of second day a dry dressing was applied with powdered antitoxin. Prophylaxis is the most important phase of this question and we can reduce danger from tetanus while looking for legislation, by education. The fight against tuberculosis has much improved since this question of education began fifteen or twenty years ago. So it will be with toy pistols and the antiseptic treatment of wounds. Education by the doctor would reduce the danger materially. Legislation will come much sooner if preceded by a campaign of education. I am a strong believer in education as a prophylactic.

Dr. F. C. Busch: The tetanus toxin is supposed to reach the cord where it exercises its specific effect, by way of the motor nerve fiber. This may account for part of the period of incubation. It has been suggested and the suggestion has been acted upon, to resect the nerves leading to the point of inoculation; this, however, would hardly seem efficacious since the poison has already been absorbed by the blood and lymph and has entered other nerve fibers, whence it may be carried to the cord.

Dr. James E. King: My interest in tetanus centers around catgut as a source of infection. The possibility of its being an infecting agent is evident. Prepared from the intestinal tract of the sheep the possibility of the spores and bacilli being incorporated in its substance, it only requires the absorption of the catgut in a wound to liberate the germ and spore under the best conditions to favor the development. Several years ago an epidemic of tetanus broke out in one of the western cities. It occurred only in operative cases and investigation discovered that it was in those cases where the catgut, prepared by a certain manufacturer, had been used. Dr. McKee having mentioned my connection with his case I feel at liberty to speak of it further. I had his case under my care during his absence; it developed the disease in a typical manner. The patient had done so nicely that for several days I had not seen her. A high pulse and a tendency to a set jaw prompted the interne to ask me to see her. I learned that the day preceding she had complained of a "stiff neck," but the bed being near a window it was supposed to be a cold and no importance attached to it. The following day when I saw her the diagnosis was very simple. Antitoxin and carbolic acid were both used in generous dose, but without effect. I took this case to be one of catgut infection. Experimental laboratory work easily finds the agent that will kill spore and germ, but in preparing catgut the strength of the catgut must be maintained and the agents and chemicals that will destroy the germ will too often destroy the usefulness of the suture. I have not found any record of experiments with catgut to determine the possibility of destroying the tetanus spore and, personally, I feel that there is a wide and useful field for some one to work in.

One manufacturer comes nearer to a perfect sterilisation of catgut than any of the methods I know anything about. The catgut to be prepared is first sterilised to kill what germs there may be. The catgut then passes through a process by which any spores are supposed to be developed and then resterilised to destroy the bacilli so produced. It is the resistance of the spore that renders killing of the tetanus germ difficult. Of the simpler methods I should suspect that the iodine method gives the best chance of killing the tetanus germ in catgut.

Dr. L. Bradley Dorr: I have seen two cases of gunshot wound causing tetanus,—one on the hand and another being an abrasion on side of neck; the first, the seventh day, and the second the eighth. Antitoxin and carbolic acid were used, and both patients recovered.

A doctor from Colden telephoned me sometime ago that he had a case of tetanus infection from a vaccine wound. By early treatment the patient got well also.

Dr. Grover W. Wende: I have had no experience with tetanus except while studying under Professor Peck in the hospital at Prague. At this time there occurred an epidemic in the Lying-in Hospital. Many of the patients died, including a couple of nurses and an interne, representing all those infected with the disease. There were a few cases in the German Hospital of which one recovered through the use of the serum. Epidemics of tetanus have occurred in this country along the shores of New Jersey and Long Island.

Dr. Bentz, in closing, said: Escharotics, as silver nitrate, irritate the tissues and favor the formation of a crust, which protects the tetanus germs in their growth. Antiseptic treatment would be effective if the conditions of the wound were such that the antiseptics reached the organisms; mixed infection then would not occur and tetanus would not develop. Carbolic acid is destructive to the toxin; iodine weakens it.

As an immunising dose, in suspicious wounds 10 c.c. of the antitoxin (Mulford's) should be injected in the region of the wound. The dry antitoxin has no effect when applied superficially.

Breiger and Boer have isolated antitoxin from the serum by the methods used to obtain the toxin.

Some parts of Long Island and New Jersey have become notorious for the number of cases of tetanus developing after trifling injuries. Cases are fairly common in New York city.

Experimentally, when tetanus toxin is mixed with emulsion of brain tissue, it is neutralised. When we consider that tetanus organisms are found in the intestinal discharges of herbivorous animals, we can understand the possibility and probability of contamination of material used in the manufacture of catgut. The cases that do occur from catgut are explained by faulty sterilisation of the catgut before operation. Vaillard says, if spores are rendered free from toxin, by being kept for a time at 80° C. and then injected into animals, death does not occur; but if lactic acid, or traumatic conditions of tissue about the wound, or mechanical irritants, as foreign bodies, or pus cocci be introduced along with spores or bacilli death results. This explains how the disease occurs naturally.

Kitasato believes that in the natural infection in man, the presence of tetanus bacilli or their spores along with foreign material is necessary.

Some poisons have a selective influence on the nervous system. In tetanus the toxin affects particular groups of neurons. The fact that lockjaw nearly always occurs first, shows that the poison selects the motor nucleus of the fifth nerve.

The poison in syphilis affects certain neurons producing the Argyll-Robertson pupil. In rabies the medulla is especially affected. The mortality of cases, in which antitoxin was injected intracerebrally is high. The method is dangerous.

The differential diagnosis between strychnia poisoning and tetanus—in the former, the history of the case, the rapidity of onset of symptoms, the complete relaxation between the spasms, and the late involvement of the jaw will indicate strychnia poisoning. In tetanus the symptoms appear late, the first symptom is stiffness of the muscles of mastication, and a spastic condition of the muscles near the wound.

The period of inoculation varies with the history of injury. It is doubtful whether any idiopathic cases exist; there must be a wound somewhere.

One point in diagnosis is important. Tetanus toxin is eliminated in the urine, also is found in cerebrospinal fluid. The injection of cerebrospinal fluid or urine from cases of the disease into susceptible animals, e. g., a white mouse, may be used as a test.

